# Supplementary material for: Urban modeling of shrinking cities through Bayesian network analysis using economic, social, and educational indicators: Case of Japanese cities
Source: PLoS One. 2023 Apr 10;18(4):e0284134. doi: 10.1371/journal.pone.0284134 (PMC10085021; doi:10.1371/journal.pone.0284134)
Supplement: S1 Table — The table below shows the 259 indicators with their average, standard deviation score, standardized total effect, mutual information, and p-value. (PDF) [file pone.0284134.s001.pdf]

S1 Table. Results of the Economic, Social, and Educational Indicators. The table bellow showas the 259 indicators with their average, standard deciation score, standardized total effect, mutual information, and p-value.

Indicators	Average	Standard Deviation	STE	MI	p-value
● Total number of elementary school students	2254.07	4067.73	0.10	0.00	0.01 *
● Number of public elementary school students	2230.51	3970.59	0.10	0.00	0.00 **
● Percentage of students enrolled in public elementary school	99.78	1.48	-0.02	-0.06	1.00
● Percentage of students enrolled in private elementary school	0.22	1.48	0.02	0.06	0.99
● Total number of junior high school students	1188.33	2108.25	0.09	0.00	0.02 *
● Number of public junior high school students	1141.21	1945.68	0.11	0.00	0.00 **
● Percentage of students enrolled in public junior high school	98.55	7.16	-0.03	-0.01	0.98
● Percentage of students enrolled in private junior high school	1.15	4.65	0.03	0.02	0.95
● Total number of junior high school graduates	418.64	728.40	0.10	0.00	0.01 *
● Number of students who go on to high school after graduating from junior high school	411.20	713.68	0.10	0.00	0.01 **
● Number of students who get a job after graduating from junior high school	1.56	3.81	0.07	0.07	0.53
● Percentage of students who go on to high school after graduating from junior high school	98.27	5.66	0.00	0.00	1.00
● Percentage of students who get a job after graduating from junior high school	0.31	0.62	-0.01	-0.03	1.00
● Male population aged over 15	18712.48	49658.88	0.08	0.00	0.02 *
● Male population of graduates aged over 15	17203.29	45082.00	0.08	0.00	0.02 *
● Male population of graduates from high school aged over 15	7993.41	18118.02	0.08	0.00	0.03 *
● Male population of graduates from junior or technical colledge aged over 15	1064.84	3563.99	0.08	0.00	0.02 *
● Male population of graduates from university or graduate school aged over 15	2956.86	14434.78	0.06	0.00	0.34
● Female population aged over 15	20400.30	50842.15	0.08	0.00	0.07
● Female population of graduates aged over 15	18973.48	46894.30	0.08	0.00	0.07
● Female population of graduates from high school aged over 15	8923.87	21807.34	0.08	0.00	0.05
● Female population of graduates from junior or technical colledge aged over 15	2641.80	9455.65	0.07	0.00	0.15
● Female population of graduates from university or graduate school aged over 15	993.37	5289.93	0.06	0.00	0.23
● Population aged over 15	332306.45	166629.39	#N/A	#N/A	#N/A
● Population of employee aged over 15	193909.68	100371.11	#N/A	#N/A	#N/A
● Population of employee who graduated aged over 15	186606.45	97282.22	#N/A	#N/A	#N/A
● Population of employee who graduated from high school aged over 15	85551.61	45920.31	#N/A	#N/A	#N/A
● Population of employee who graduated from vocational school aged over 15	21283.87	11561.38	#N/A	#N/A	#N/A
● Population of employee who graduated from junior or technical colledge aged over 15	16487.10	9252.49	#N/A	#N/A	#N/A
● Population of employee who graduated from university or graduate school aged over 15	41629.03	20378.46	#N/A	#N/A	#N/A
● Population of unemployee aged over 15	138400.00	68031.95	#N/A	#N/A	#N/A
● Population of unemployee who graduated aged over 15	113483.87	57639.58	#N/A	#N/A	#N/A
● Population of unemployee who graduated from high school aged over 15	50490.32	26925.36	#N/A	#N/A	#N/A
● Population of unemployee who graduated from vocational school aged over 15	6764.52	3382.56	#N/A	#N/A	#N/A

● Population of unemployee who graduated from junior or technical colledge aged over 15	8290.32	4147.48	#N/A	#N/A	#N/A
● Population of unemployee who graduated from university or graduate school aged over 15	8519.35	5000.03	#N/A	#N/A	#N/A
● Percentagre of employee to unemployee	64.62	3.14	#N/A	#N/A	#N/A
● Percentagre of employee	58.19	2.72	#N/A	#N/A	#N/A
● Population aged 0-4	1596.14	3008.13	0.10	0.00	0.01 **
● Number of children on waiting list for daycare centers	2.46	14.19	0.05	0.01	0.89
● Number of kindergartens	5.17	8.98	0.08	0.04	1.00
● Number of students enrolled in kindergarten	435.38	1034.20	0.08	0.00	0.12
● Number of childcare centers	9.43	13.87	0.10	0.03	0.02 *
● Number of public childcare centers	4.62	6.21	0.08	0.05	0.39
● Number of private childcare centers	4.81	9.75	0.09	0.03	0.10
● Number of children enrolled in childcare centers	788.78	1325.61	0.11	0.00	0.00 **
● Number of children enrolled in public childcare centers	336.65	513.14	0.07	0.00	0.63
● Number of children enrolled in private childcare centers	452.13	965.94	0.09	0.00	0.04 *
● Number of people checked out from public libraries	51052.34	226734.77	0.08	0.00	0.05
● Number of children checked out from public libraries	7393.50	20593.55	0.07	0.00	0.42
● Number of books checked out from public libraries	183807.63	413096.76	0.08	0.00	0.14
● Number of cultural centers	0.86	1.17	0.07	0.20	0.70
● Number of community centers	9.44	15.63	0.04	0.01	0.92
● Number of libraries	1.41	1.97	0.07	0.13	0.49
● Total number of children's centers	1.61	3.46	0.04	0.04	0.86
● Number of public children's centers	1.02	2.23	0.03	0.05	1.00
● Number of private children's centers	0.59	2.64	0.02	0.04	1.00
● Number of establishments in all industries	2105.95	3698.62	0.08	0.00	0.12
● Number of establishments in education and learning support industry	80.93	135.42	0.09	0.00	0.06
● Percentage of establishments in education and learning support industry	4.02	1.30	#N/A	#N/A	#N/A
● Life protection rate	18.51	10.48	#N/A	#N/A	#N/A
● Number of assisted persons who receive public assistance	261331.04	165397.29	#N/A	#N/A	#N/A
● Number of educational assistants who receive public assistance	7862.87	6365.43	#N/A	#N/A	#N/A
● Pecentage of educational assistants who receive public assistance	2.86	0.77	#N/A	#N/A	#N/A
● Number of students per educational computer in schools	5.12	2.48	0.21	0.31	0.00 **
● Number of students per educational computer in elementary schools	5.84	3.32	0.16	0.18	0.00 **
● Number of students per educational computer in junior high schools	4.66	2.61	0.25	0.35	0.00 **
● Number of students per educational computer in high schools	3.87	2.88	#N/A	#N/A	#N/A
● Percentage of schools connected to superfast Internet (fiber optic line)	54.07	43.18	0.05	0.00	0.99
● Percentage of elementary schools connected to superfast Internet (fiber optic line)	54.01	44.22	0.06	0.00	0.98
● Percentage of junior high schools connected to superfast Internet (fiber optic line)	54.54	45.68	0.05	0.00	0.99
● Percentage of high schools connected to superfast Internet (fiber optic line)	55.63	49.68	#N/A	#N/A	#N/A
● Percentage of schools connected to superfast Internet (Over 30 Mbps lines)	60.41	36.71	0.02	0.00	1.00
● Percentage of elementary schools connected to superfast Internet (Over 30 Mbps lines)	59.87	39.11	0.03	0.00	1.00
● Percentage of junior high schools connected to superfast Internet (Over 30 Mbps lines)	61.64	42.14	0.02	0.00	1.00
● Percentage of high schools connected to superfast Internet (Over 30 Mbps lines)	57.29	49.17	#N/A	#N/A	#N/A
● Number of elementary school teachers	158.94	230.42	0.11	0.00	0.00 **
● Number of elementary school children	2298.96	4126.59	0.10	0.00	0.00 **
● Number of children per teacher in elementary school	11.86	4.16	0.47	0.41	0.00 **
● Number of junior high school teachers	95.91	142.27	0.10	0.00	0.01 **

●	Number of junior high school students	1215.95	2135.73	0.09	0.00	0.02	*
●	Number of children per teacher in junior high school	10.57	3.82	0.36	0.34	0.00	**
●	Number of elementary schools with combined classes	101.45	154.61	0.11	0.00	0.00	**
●	Number of combined classes in elementary school	4.05	6.23	-0.13	-0.07	0.00	**
●	Percentage of combined classes in elementary school	7.71	12.64	-0.27	-0.08	0.00	**
●	Number of junior high schools with combined classes	43.90	70.20	0.10	0.01	0.02	*
●	Number of combined classes in junior high school	0.12	0.54	-0.06	-0.42	0.44	
●	Percentage of combined classes in junior high school	0.81	4.66	-0.09	-0.08	0.08	
●	Number of high schools	2.16	3.29	0.08	0.09	0.28	
●	Number of universities	0.28	1.16	0.05	0.17	0.93	
●	Number of junior colleges	0.16	0.65	0.04	0.22	0.92	
●	Total population	41553.78	73669.40	0.09	0.00	0.03	*
●	Population aged 0-14	5326.07	9660.38	0.10	0.00	0.00	**
●	Population aged 15-64	25137.91	45824.54	0.09	0.00	0.07	
●	Population over 65	10905.42	17716.53	0.09	0.00	0.02	*
●	Percentage of population aged 0-14	12.02	1.93	0.51	0.98	0.00	**
●	Population of over 65	30.16	6.26	-0.70	-0.41	0.00	**
●	Population moving into municipality	4876.72	8680.14	0.09	0.00	0.07	
●	Population moving out of municipality	5141.99	9028.70	0.09	0.00	0.02	*
●	Percentage population moving into municipality	10.74	4.02	0.01	0.01	1.00	
●	Percentage population moving out of municipality	11.76	2.91	0.01	0.01	1.00	
●	Average age of unmarried males	34.20	2.76	-0.43	-0.56	0.00	**
●	Average age of unmarried female	31.75	3.05	-0.26	-0.34	0.00	**
●	Total population (census)	41298.58	73444.45	0.09	0.00	0.04	*
●	Male population	19931.84	35429.93	0.10	0.00	0.00	**
●	Female population	21366.73	38037.03	0.09	0.00	0.05	
●	Number of births	327.11	627.12	0.09	0.00	0.01	*
●	Number of deaths	451.09	695.46	0.09	0.00	0.07	
●	Natural population change	-123.99	162.00	0.04	0.00	0.09	
●	Natural population change rate	-0.59	0.46	0.57	4.56	0.00	**
●	Population moving into municipality	1264.97	2624.32	0.09	0.00	0.07	
●	Population moving out of municipality	1590.77	3010.65	0.09	0.00	0.02	*
●	Social population change	-255.49	392.38	#N/A	#N/A	#N/A	
●	Social population change rate	-0.72	0.65	#N/A	#N/A	#N/A	
●	Number of divorces	77.55	146.29	0.08	0.00	0.27	
●	Male life expectancy	78.28	0.95	0.00	-0.02	1.00	
●	Female life expectancy	85.70	0.70	0.01	0.04	1.00	
●	total fertility rate	1.45	0.18	0.08	1.50	0.73	
●	Fertility rate of mothers aged 15-19	5.86	1.90	0.01	0.02	1.00	
●	Fertility rate of mothers aged 20-24	54.92	17.06	0.02	0.01	1.00	
●	Fertility rate of mothers aged 25-29	102.62	13.87	0.04	0.01	1.00	
●	Fertility rate of mothers aged 30-34	87.83	9.04	0.02	0.01	1.00	
●	Fertility rate of mothers aged 35-39	35.74	4.71	0.01	0.01	1.00	
●	Fertility rate of mothers aged 40-44	4.74	0.99	0.01	0.03	1.00	
●	Fertility rate of mothers aged 45-49	0.12	0.07	0.00	0.09	1.00	
●	Average age of father's first child born	31.80	0.42	#N/A	#N/A	#N/A	
●	Average age of mother's first child born	29.83	0.36	#N/A	#N/A	#N/A	
●	Population change rate	-5.64	3.67	#N/A	#N/A	#N/A	
●	Population change rate aged 0-14	-13.13	7.04	0.73	0.38	0.00	**
●	Population change rate aged over 65	5.04	8.07	0.30	0.14	0.00	**
●	Number of households	15648.84	29466.59	0.08	0.00	0.13	
●	Number of nuclear households	11534.87	20285.28	0.10	0.00	0.01	**
●	Percentage of nuclear households	77.29	6.74	-0.03	-0.02	1.00	
●	Number of single-mother households	1245.72	2412.26	0.08	0.00	0.18	
●	Percentage of single-mother households	7.60	1.35	0.03	0.08	1.00	
●	Number of single-father households	214.34	381.72	0.09	0.00	0.02	*
●	Percentage of single-father households	1.42	0.31	0.01	0.12	1.00	
●	Number of households with three generations living together	2163.07	3836.08	0.11	0.00	0.00	**
●	Percentage of households with three generations living together	14.73	7.85	0.00	0.00	1.00	
●	Number of households living in housing	15153.51	28145.05	0.08	0.00	0.11	

Number of households living in owner-occupied housing	10700.10	17380.10	0.10	0.00	0.02	*
Percentage of households living in owner-occupied housing	77.92	11.02	-0.02	-0.01	1.00	
Number of households living in public housing	867.46	2066.96	0.06	0.00	0.51	
Percentage of households living in public housing	6.84	6.04	-0.12	-0.07	0.01	**
Standard price of residential land	24205.65	21818.72	#N/A	#N/A	#N/A	
Number of land transactions	410.69	666.20	0.09	0.00	0.07	
Area of land transactions	81.52	159.11	-0.03	0.00	0.98	
Gross floor area per houses	122.50	24.53	-0.01	0.00	1.00	
Number of houses for resident households	24077.24	34345.08	0.06	0.00	0.78	
Number of owned houses	16811.23	20583.27	0.06	0.00	0.70	
Number of rented houses	6862.80	13189.79	0.05	0.00	0.75	
Percentage of owned houses to resident households	76.13	10.25	-0.02	-0.01	1.00	
Percentage of rented houses to resident households	22.89	9.82	0.02	0.01	1.00	
Total number of houses	24446.54	34540.50	0.06	0.00	0.68	
Number of homes located more than 500 meters from the nearest childcare center	19227.97	23674.43	0.07	0.00	0.36	
Percentage of homes located more than 500 meters from the nearest childcare center	85.01	9.98	-0.05	-0.02	0.96	
Number of homes located more than 500 meters from the nearest elementary school	21631.84	28594.30	0.06	0.00	0.54	
Percentage of homes located more than 500 meters from the nearest elementary school	91.51	5.76	0.00	0.00	1.00	
Number of homes located more than 1 km from the nearest junior high school	18615.54	22694.24	0.07	0.00	0.28	
Percentage of homes located more than 1 km from the nearest junior high school	82.52	11.03	-0.02	-0.01	1.00	
Number of people receiving health checkups	4108.43	6358.26	0.06	0.00	0.79	
Number of people receiving medical checkups for malignant neoplasms	1265.49	2944.80	0.04	0.00	0.98	
Percentage of people receiving medical checkups for malignant neoplasms	5.44	12.19	-0.01	0.00	1.00	
Number of persons eligible for vaccination against measles or rubella	1546.13	2741.11	0.10	0.00	0.00	**
Number of persons vaccinated against measles or rubella	1348.54	2381.10	0.10	0.00	0.00	**
Percentage of persons vaccinated against measles or rubella	159.04	2557.80	-0.09	0.00	0.25	
Number of persons eligible for vaccination against influenza	10985.17	18048.07	0.09	0.00	0.04	*
Number of persons vaccinated against influenza	5710.52	9124.25	0.08	0.00	0.14	
Percentage of persons vaccinated against influenza	53.96	12.14	-0.09	-0.03	0.18	
Number of pregnancies reported	337.59	660.23	0.09	0.00	0.01	*
Number of pregnant receiving medical checkups	450.56	947.80	0.08	0.00	0.06	
Number of hospitals	3.37	6.24	0.07	0.04	0.32	
Number of medical clinics	31.49	65.08	0.08	0.00	0.14	
Number of dental clinics	19.64	41.18	0.08	0.01	0.08	
Number of hospital beds	589.44	1223.15	0.07	0.00	0.57	
Number of beds in medical clinics	59.15	135.77	0.06	0.00	0.74	
Number of doctors	84.88	213.70	0.05	0.00	0.81	
Number of dentists	28.05	70.44	0.07	0.00	0.16	
Number of traffic accidents	234.57	563.01	0.08	0.00	0.02	*
Number of urban parks	28.78	84.14	#N/A	#N/A	#N/A	
Nominal gross production in prefecture	4777884.50	1776053.54	#N/A	#N/A	#N/A	
Percentage change of nominal gross production in prefecture	-2.79	0.77	#N/A	#N/A	#N/A	
Real gross production in prefecture	5169132.00	2030687.64	#N/A	#N/A	#N/A	
Percentage change of real gross production in prefecture	-2.28	1.13	#N/A	#N/A	#N/A	
Per capita income of prefectural citizens	2809.96	181.00	#N/A	#N/A	#N/A	
Percentage change of per capita income of prefectural citizens	-4.67	0.95	#N/A	#N/A	#N/A	
Per capita compensation for employees in prefecture	4961.20	250.33	#N/A	#N/A	#N/A	
Percentage change of per capita compensation for employees in prefecture	-2.86	0.19	#N/A	#N/A	#N/A	

Financial Strength Index	0.46	0.26	0.29	4.09	0.00 **
Ratio of ordinary income and expenditure	88.43	6.21	0.06	0.04	0.92
Ratio of real public debt cost	14.46	4.42	-0.10	-0.08	0.27
Future burden ratio	103.98	62.65	#N/A	#N/A	#N/A
Labor force	22116.39	37983.54	0.09	0.00	0.02 *
Number of employees	20822.90	35660.98	0.09	0.00	0.02 *
Number of unemployees	1293.49	2404.63	0.07	0.00	0.26
Number of all establishments	2166.98	3859.03	0.08	0.00	0.11
Number of establishments in agriculture and forestry	17.67	21.50	0.04	0.01	0.97
Number of establishments in the fishing industry	2.71	6.91	-0.05	-0.02	0.93
Number of establishments in mining / quarrying / gravel extraction industries	1.65	2.81	0.04	0.05	0.97
Number of establishments in the construction industry	226.88	359.27	0.11	0.00	0.00 **
Number of establishments in the manufacturing industry	195.36	390.13	0.09	0.00	0.01 *
Number of establishments in the electricity / gas / heat supply / water supply industries	4.03	5.16	0.07	0.05	0.59
Number of establishments in the information and communication industry	13.24	37.71	0.06	0.01	0.53
Number of establishments in transportation and postal services	48.67	90.76	0.09	0.00	0.05
Number of establishments in wholesale and retail trade	551.46	996.91	0.08	0.00	0.15
Number of establishments in the financial and insurance industry	31.08	67.57	0.07	0.00	0.43
Number of establishments in the real estate and goods rental industry	109.72	262.35	0.07	0.00	0.33
Number of establishments in academic research and professional-technical services	63.69	144.18	0.07	0.00	0.25
Number of establishments in accommodation and food service industory	260.10	477.13	0.08	0.00	0.23
Number of establishments in the household services and entertainment industry	189.45	329.78	0.08	0.00	0.18
Number of establishments in education and learning support industry	80.78	135.23	0.09	0.00	0.06
Number of establishments in medicine and welfare	128.43	236.70	0.09	0.00	0.06
Number of establishments in multi-service businesses industory	19.02	23.78	0.09	0.01	0.17
Number of establishments in other service industries	137.89	238.26	0.08	0.00	0.21
Number of establishments in public service	20.89	22.57	0.08	0.01	0.41
Shipment of manufactured products	12364669.35	26643283.38	#N/A	#N/A	#N/A
Annual Commercial Sales	90505.99	258829.04	0.06	0.00	0.50
Employment rate	94.52	2.09	-0.04	-0.08	0.96
Unmployment rate	5.48	2.09	0.04	0.08	0.96
Average weekly working hours	41.58	1.87	-0.07	-0.16	0.50
Average weekly hours worked of males	45.12	2.03	0.00	0.01	0.92
Average weekly hours worked of female	36.84	2.44	-0.10	-0.16	0.07
Number of workers in primary industry	1774.62	2038.35	0.06	0.00	0.74
Number of workers in secondary industry	5935.89	9999.90	0.11	0.00	0.00 **
Number of workers in tertiary industry	12903.64	24379.71	0.08	0.00	0.17
Percentage of workers in primary industry	14.94	10.53	-0.20	-0.07	0.00 **
Percentage of workers in secondary industry	27.97	8.18	0.09	0.04	0.17
Percentage of workers in tertiary industry	57.09	9.58	0.12	0.04	0.02 *
Number of commuters to other municipalities	6337.84	10918.21	0.14	0.00	0.00 **
Number of workers	193909.68	100371.11	#N/A	#N/A	#N/A
Number of self-employed people	19361.29	10223.04	#N/A	#N/A	#N/A
Number of family employees	5164.52	3032.66	#N/A	#N/A	#N/A
Number of employees	169087.10	88754.93	0.09	0.00	0.02 *
Number of company directors	11248.39	5356.30	#N/A	#N/A	#N/A
Number of permanent employees	136306.45	72476.56	#N/A	#N/A	#N/A
Number of temporary employees	17574.19	9534.25	#N/A	#N/A	#N/A

● Number of daily hired employees	3964.52	2620.63	#N/A	#N/A	#N/A
● Percentage of permanent employees to employees	60.85	2.48	#N/A	#N/A	#N/A
● Percentage of part-time workers to employees	22.36	2.05	#N/A	#N/A	#N/A
● Number of employees among workers	15734.55	28402.70	0.09	0.00	0.04 *
● Number of temporary employees among employees	2532.06	4300.63	0.09	0.00	0.08
● Percentage of temporary employees to employees	18.69	5.09	-0.17	-0.12	0.00 **
● Number of households with married couples	9889.05	17180.76	0.10	0.00	0.01 *
● Number of dual-earner couples among married couples	4748.60	7657.50	0.12	0.00	0.00 **
● Percentage of dual-earner couples to married couples	51.18	8.27	-0.07	-0.03	0.54
● Number of households with married couples and children	6133.59	10783.52	0.10	0.00	0.00 **
● Number of dual-earner couples to married couples and children	3357.11	5465.13	0.12	0.00	0.00 **
● Percentage of dual-earner couples to married couples with children	59.01	8.20	-0.09	-0.04	0.29
● Number of white-collar workers aged over 15	20832.55	35749.86	0.09	0.00	0.02 *
● Number of white-collar workers	9160.86	17796.40	0.08	0.00	0.23
● Percentage of white-collar workers	38.39	7.15	0.15	0.08	0.00 **
● Taxable income	50982.01	101468.67	0.08	0.00	0.08
● Number of taxpayers	17477.43	32347.34	0.09	0.00	0.05 *
● Assets per household	28999.14	11707.11	#N/A	#N/A	#N/A
● Current savings balance per household	14162.25	5343.90	#N/A	#N/A	#N/A
● Debt per household	3852.96	2089.96	0.02	0.00	1.00
● Total land area	247.14	257.33	-0.08	0.00	0.41
● Habitable Area	72.33	74.32	#N/A	#N/A	#N/A

Note. STE, Standardized Total Effect; MI, Mutual Information; *, p <0.05; **, p<0.01
