# Supplementary material for: Urban modeling of shrinking cities through Bayesian network analysis using economic, social, and educational indicators: Case of Japanese cities
Source: PLoS One. 2023 Apr 10;18(4):e0284134. doi: 10.1371/journal.pone.0284134 (PMC10085021; doi:10.1371/journal.pone.0284134)
Supplement: Attachment — Submitted filename: 3_Response to Reviewers_20230308.pdf [file pone.0284134.s002.pdf]

PONE-D-22-35408

Dear Reviewers and Editor

Haruka KATO

I appreciate the time and effort you have dedicated to providing insightful feedback on strengthening my manuscript. Thus, it is with great pleasure that I re-submit my article for further consideration. I have incorporated changes that reflect the detailed suggestions you have graciously provided. I also hope that my edits and the responses I provide below satisfy the issue and concern you have noted. The revised manuscript is marked in red font with tracked change.

General Comment (Reviewer #1)

> The authors analyzed the impacts of economic, social, and educational factors on population decline in Japanese shrinking cities. The disadvantages of this research are that the research content is simple, the structure is irrational, and the innovation is insufficient. I focus here only on some points, which are hopefully easy for the authors to take into account in the revision.

Author Response:

I appreciate your evaluation and meaningful comments on our manuscripts. According to your comments, I revised the manuscript as follows:

Comment No.1 (Reviewer #1)

> (1) Line 33-34, Fig.1 is not clear enough, and I do not understand population change rate in 2010. What does it mean?

Author Response:

I appreciate your comments on the meaning of the population change rate (PCR). PCR is the percentage of change from the population in 2005 (P_{2005}) to the population in 2010 (P_{2010}). I also added the equation of the PCR. According to the revision of abbreviations (PCR), the word of PCR was corrected throughout the manuscript.

Manuscript Revision:

Ln 21-22	In Fig 1, PCR is the percentage of change from population in 2005 (P_{2005}) to population in 2010 (P_{2010}), as calculated in Equation (1).
Ln 29	$$PCR = \frac{P_{2010} - P_{2005}}{P_{2005}} \times 100 \quad (1)$$
Ln 46-47	This study aims to clarify the indicators that affect the PCR by modeling shrinking cities. For the analysis, this study uses the case of Japanese cities.
Ln 116-117	For the analysis, this study set the indicator of PCR as the target variable; Bayesian network analysis revealed the total effect (TE) and correlation of indicators on the PCR.
Ln 120-121	The standardized total effect (STE) normalized the TE by taking into account the ratio between the standard deviations of the indicators (σ_x) and the target indicator (PCR) (σ_y)

	[33].
Ln 124-125	Correlation analysis was based on mutual information (MI), defined as the difference between the marginal entropy H(T) of the target indicator (PCR) and its conditional entropy H(T|X). The binary mutual information (BMI) is the amount of information brought by each indicator to the knowledge of the state of the target indicator (PCR) compared to an unconnected network [29].
Ln 149-150	The lower graph shows the network around the PCR.
Ln 161-163	The PCR is also found to be affected by educational indicators, with relevant factors including "number of children per teacher in elementary school" (STE = 0.47), "percentage of combined classes in elementary school" (STE = -0.27), and "number of students per educational computer in schools" (STE = 0.21).

Comment No.2 (Reviewer #1)

(2) Line 37, 'However, these risks have not been identified systematically.', add more details.

Author Response:

I appreciate your comment on the unclear sentence. In the previous manuscript, the risk means the cause of population decline. However, the message was inaccurate. Therefore, I revised the sentence in more detail.

Manuscript Revision:

Ln 26-28	However, there is a cascading and complex relationship between economic and social factors within the context of population decline, and researchers have yet to systematically elucidate the factors affecting population decline.

Comment No.3 (Reviewer #1)

(3) Sec Literature Review, conlude it and highlight the innovation.

Author Response:

I appreciate your meaningful comments on the literature review. According to your comments, I concluded the previous findings and highlighted the novelty of this study.

Manuscript Revision:

Ln 61	The novelty of this study is to develop an urban statistical model of shrinking cities by Bayesian network analysis.
Ln 80-84	Despite the important contributions of these past studies, they failed to elucidate the complex and cascading relationships of factors related to population decline. Accordingly, the originality of this study lies in its extension of these prior pieces of evidence by developing an urban statistical model of shrinking cities using a dataset comprising economic, social, and educational indicators, and in its aim to identify the

	factors influencing PCR.

Comment No.4 (Reviewer #1)

(4) Sec Economic, Social, and Educational Indicators, why choose this these indicators, explain it. Importantly, Is there collinearity?

Author Response:

I appreciate your comments on our data. The reason why chooses this dataset is that it covers most city-level government statistics that are available publicly in Japan. As you are concerned, the data might have collinearity. Therefore, I applied the Bayesian network analysis to avoid collinearity risk. I added the explanation in the revised manuscript.

Manuscript Revision:

Ln 91-92	This study used this dataset because it covers most publicly available city-level government statistics in Japan
Ln 108-110	It also enables researchers to obtain robust models that avoid collinearity risk [33]. It is important for the current study that that the analytical process of choice can avoid collinearity risk because 259 indicators are under scrutiny.

Comment No.5 (Reviewer #1)

(5) Sec Discussions, this part should be improved. Does urban climate change affect city shrinkage and population. Some important references as follows:

1) Understanding seasonal contributions of urban morphology to thermal environment based on boosted regression tree approach, Building and Environment, 2022, 2022, 226: 109770, doi: 10.1016/j.buildenv.2022.109770.

2) Contribution of urban functional zones to the spatial distribution of urban thermal environment, Building and Environment (2022), doi: 10.1016/j.buildenv.2022.109000.

3) Modelling spatial distribution of fine-scale populations based on residential properties, International Journal of Remote Sensing (2019), doi: https://doi.org/10.1080/01431161.2019.1579387.

4) Exploring thermal comfort of urban buildings based on local climate zones, Journal of Cleaner Production (2022), doi: https://doi.org/10.1016/j.jclepro.2022.130744.

5) The impact of urban renewal on land surface temperature changes: A case study in the main city of Guangzhou, China. Remote Sensing (2020), https://doi.org/10.3390/rs12050794.

Author Response:

I appreciate your comments on the discussion. As you suggested, population decline might correlated with climate change on the earth scale. Your comment made me aware of the need to add a discussion. I added the discussion with some additional references.

Manuscript Revision:

Ln 222-232	It should be noted that this study was conducted in Japan, a country characterized by a stable climate. However, in some countries worldwide, including those in the Global South, global warming issues might cause population decline. Particularly, researchers show that there are correlations between the urban population, the built environment, and land surface temperatures [38–40], and that land surface temperatures are influenced more by the built than natural environment [41]. Another study shows that the relationship between these factors and urban temperature is nonlinear [42]. These pieces of evidence suggest that populations might begin to decline as temperatures exceed a threshold. Specifically, the natural disasters caused by global warming, such as hurricanes and floods, have been reported to be related to emerging refugee issues [43]. Thus, future researchers attempting to use methods and conduct analyses similar to those of the current study in the context of other countries may need to also consider the impact of global warming. Scholars have also remarked that research networks enabling the interaction between research and practice play an essential role in global research regarding shrinking cities [44].
Ln 335-347	38. Chen Y, Yang J, Yang R, Xiao X, Xia J (Cecilia). Contribution of urban functional zones to the spatial distribution of urban thermal environment. Build Environ. 2022;216: 109000. doi:10.1016/j.buildenv.2022.109000<br>39. Han D, Yang X, Cai H, Xu X, Qiao Z, Cheng C, et al. Modelling spatial distribution of fine-scale populations based on residential properties. Int J Remote Sens. 2019;40: 5287–5300. doi:10.1080/01431161.2019.1579387<br>40. Qiao Z, Liu L, Qin Y, Xu X, Wang B, Liu Z. The Impact of Urban Renewal on Land Surface Temperature Changes: A Case Study in the Main City of Guangzhou, China. Remote Sens (Basel). 2020;12: 794. doi:10.3390/rs12050794<br>41. Ren J, Yang J, Zhang Y, Xiao X, Xia JC, Li X, et al. Exploring thermal comfort of urban buildings based on local climate zones. J Clean Prod. 2022;340: 130744. doi:10.1016/j.jclepro.2022.130744<br>42. Han D, An H, Wang F, Xu X, Qiao Z, Wang M, et al. Understanding seasonal contributions of urban morphology to thermal environment based on boosted regression tree approach. Build Environ. 2022;226: 109770. doi:10.1016/j.buildenv.2022.109770<br>43. Berchin II, Valduga IB, Garcia J, de Andrade Guerra JBSO. Climate change and forced migrations: An effort towards recognizing climate refugees. Geoforum. 2017;84: 147–150. doi:10.1016/j.geoforum.2017.06.022

Comment No.6 (Reviewer #1)

(6) Sec Conclusions, conclude the main colusions of this study, remove references.

Author Response:

I appreciate your comments on the conclusion. According to your suggestion, I removed references and concluded my main conclusions sharply.

Manuscript Revision:

Ln 243-253	In conclusion, the study findings provide essential insights into the importance of education investment for children in shrinking cities. Regarding theoretical implications, this study shows that the impact of educational investment is more substantial than that of economic indicators (e.g., the financial strength index). With this in mind and considering the limitations regarding fiscal expenditures in Japan, it may be important for municipal leaders to invest in education for children in order to prevent rapid population decline. In summary, increasing the budget for education to tackle the decline in the number of children and increasing the welfare expenditure to tackle the growth in the number of older adults are likely important measures in the context of population decline because of low birth rates and aging populations.<br>In Japan, population decline is expected to continue for a long time, as immigration is low. In this context, Japanese shrinking cities need to achieve a gradual, rather than a rapid, population decline to maintain residents' abundant lifestyle. The conclusions of this study could contribute to countries other than Japan that are likely to experience future population decline because of low birth rates and aging populations.

Comment No.7 (Reviewer #1)

(7) English language should be improved.

Author Response:

I appreciate your meaningful comments on our manuscripts. According to your comments, this manuscript was improved by the English editing service. The certificate letter is attached in the last page of this response letter.

Manuscript Revision:

Ln 4-5	By modeling shrinking cities using the case of Japanese cities, this study aims to clarify the indicators that affect the population change rate.
Ln 5-6	In conclusion, this study demonstrates that social and educational indicators affect the population decline rate.
Ln 8-9	Considering the limitations in fiscal expenditures, increasing investment in education might help solve the problem of shrinking cities because of low birthrates and aging populations.
Ln 43-44	By providing systematic insights into the issues emerging due to population decline, the

	model can deliver a planning support system for municipal leaders and urban planners.
Ln 46	This study aims to clarify the indicators that affect the PCR by modeling shrinking cities.
Ln 50-51	Therefore, this study's results from Japan may contribute by providing insights and a planning support system for shrinking cities worldwide facing similar problems.
Ln 53-55	The Japanese Ministry of Education, Culture, Sports, Science, and Technology (MEXT) developed the dataset used in this study to promote research that contributes to policy formulation.
Ln 62-63	In an early study, the researchers conceptualized an urban model consisting of economic and population decline and policy changes [18].
Ln 63-65	As research on shrinking cities progressed, particularly in Europe, researchers also conceptualized an urban model in which diverse forms of decline at the global and regional levels caused population declines [19].
Ln 67-69	Therefore, to investigate the complicated relationships among these indicators, researchers need to develop both an urban statistical model that strength conceptual models of shrinking cities.
Ln 71-77	Specifically, the positive correlation between population change and industrial diversity was clarified [21]. For example, it was investigated the case of Yubari City, a single-industry mining city in Japan, well-known for suffering from rapid population decline and financial collapse because of the mining industry decline [22]. From a different perspective, researchers demonstrated the positive correlation between investment network centralities and population change in shrinking cities [23]. In addition, it was explained that development zone policies have helped curb population decline in these shrinking cities [24]. Academicians also demonstrated that shrinking cities are influenced by socioeconomic development and social indicators, including expenditure on education [25].
Ln 81-85	Accordingly, the originality of this study lies in its extension of these prior pieces of evidence by developing an urban statistical model of shrinking cities using a dataset comprising economic, social, and educational indicators, and in its aim to identify the factors influencing PCR. For the analytical methodology, this study refers to the study by Kato [28], wherein urban modeling developed by Bayesian network analysis was used to estimate future populations.
Ln 89-90	As mentioned above, this study's data come from a cross-sectional dataset of economic, social, and educational indicators by cities which MEXT collected to promote research contributing to the formulation of educational policies [16].
Ln 99-101	The indicators present in the dataset are used not only for policymaking but also for academic research [30,31], and the dataset encompasses 259 indicators in total [32]. This

	study used all 259 indicators containing numerical and nominal scale data.
Ln 139-147	The lower part of Fig 2 shows a Bayesian network focusing on PCR as the target indicator. Among social indicators, "population change rate aged 0-14" and " population aged over 65" are strongly related to the PCR. "Population aged over 65" has a negative correlation coefficient, indicating that an increase in the number of older adults results in a decrease in the PCR. Meanwhile, relevant educational indicators include "number of children per teacher in elementary schools," "number of children per teacher in junior high schools," and "number of students per educational computer in junior high schools. "Financial strength index," which serves to represent the financial strength of a local government and is the average of the values obtained by dividing the standard financial revenue amount by the standard financial demand amount over the past three years, is one of the relevant economic indicators. Finally, urban planning indicators were not found to be related to PCR.
Ln 149-150	The upper graph shows the whole network. The lower graph shows the network around the PCR. In the lower graph, numbers between links indicate the Pearson's correlation coefficient.
Ln 155-171	Table 1 shows that the most significant indicators of the PCR are the social indicators of "population change rate aged 0-14" (STE = 0.73), " population aged over 65" (STE = -0.70), "average age of unmarried men" (STE = -0.43), and "natural population change rate" (STE = 0.57). In shrinking cities with a PCR under -12.6, BMI is higher for "population change rate aged 0-14" (BMI = 0.35) and " population aged over 65" (BMI = 0.35), suggesting that these two indicators strongly correlate with increased population change. Thus, population decline in Japanese shrinking cities occurred primarily due to natural population change rather than population change because of migration.<br>The PCR is also found to be affected by educational indicators, with relevant factors including "number of children per teacher in elementary school" (STE = 0.47), "percentage of combined classes in elementary school" (STE = -0.27), and "number of students per educational computer in schools" (STE = 0.21). Therefore, population change is related to aspects of education, such as the number of teachers and educational computers.<br>Two economic indicators are shown to affect the PCR, namely "financial strength index" (STE = 0.29) and "percentage of workers in primary industry" (STE = -0.20), albeit other economic indicators such as "per capita income of prefectural citizens, and "taxable income" did not affect PCR. Hence, the analysis suggests that the PCR is more strongly influenced by social and educational indicators than economic indicators in Japan.

Many other indicators show a weak relationship with the PCR but are not listed in Table 1, including indicators related to urban facilities (e.g., "number of community centers" and "number of medical clinics,") and housing, (e.g., "number of homes located more than 500 meters from the nearest elementary school" and "number of land transactions").

Ln 178-181 Fig 3 shows the probabilistic change of shrinking cities with rapid population decline. In this study, shrinking cities with rapid population decline are those with a PCR under -12.6%, which accounted for 4.6% of shrinking cities in Japan. Among these cities, the probabilities of the social indicators of "population change rate aged 0-14 ≤ -26.3" and "natural population change rate ≤ -1.3" change from 4.1% to 52.7% and from 7.0% to 38.9%, respectively.

Ln 192-221 In conclusion, this study demonstrates that social and educational indicators affect the population decline rate by modeling shrinking cities in Japan through Bayesian network analysis. The statistical urban model of this study indicates the cascading and complex relationships between the factors affecting population decline in shrinking cities. While the results regarding social indicators are in line with those of previous research [25-27], the current findings are still novel and unexpected; particularly, the study shows that educational indicators have a more substantial impact than economic indicators such as the financial strength index. Previous studies have pointed to the impact of economic decline on shrinking cities [21,23]; this occurs when people migrate out of cities as local industry declines and jobs are lost. Meanwhile, the results of this study demonstrate that population decline in Japanese shrinking cities is not because of such outflow decline related to migration but because of a natural decline related to low birthrates and aging populations.

This reality of shrinking cities in Japan might explain why children's education have a stronger impact on the PCR in this context. Parents might feel more comfortable having and raising children if city governments value educational support. The educational support includes not only teachers but also educational computers. This result is consistent with those of prior research showing that those who continue to live in shrinking cities have been found to value the attractions of the city, including social connections, and economic activities in the city [36].

Still, this relevance of educational indicators might be unique to Japan, which has experienced a declining population due to its low birthrates. In Japan, education accounts for a low percentage of national and administrative fiscal expenditures, primarily because social security expenditures for older adults are a heavy burden [37]. However, when considering the current results and the fiscal expenditure limitations in Japan, the

	suggestion is that increasing investment in education for children might help solve the problem of shrinking cities.<br>The results also suggest that urban planning indicators —including "number of community centers," "number of medical clinics," "number of homes located more than 500 meters from the nearest elementary school," and "number of land transactions"— do not directly affect population decline rates in Japanese shrinking cities. Further, based on the low STE scores for these indicators, they only indirectly affect the population decline rate. This result is surprising when seen against studies that analyzed shrinking cities in the context of urban planning [27,28]. However, these findings do not imply that urban planning has no impact on the population decline rate. Urban planners are still suggested to continue to implement urban planning strategies for shrinking cities. At the same time, considering that governments have limited financial resources, the results also suggest that it might not be necessary for governments to prioritize urban planning as a target for tackling population decline in shrinking cities. Instead, it might be better to effectively maintain and manage, not build and construct, the range of urban planning-related factors that were developed during the city's population growth period. Overall, urban planning in shrinking cities remains an important topic that needs to be considered more in the future.
Ln 235-240	Because population decline is a long-term urban phenomenon, this dataset still allowed for the provision of novel conclusions. Considering this limitation, researchers could endeavor to combine the most recent and past datasets comprising economic, social, and educational indicators of cities into time series data, and then analyze the urban model developed in this study using this time series dataset. Such analysis may help us investigate future scenarios based on the urban model developed in this study.

General Comment (Reviewer #2)

The author used bayesian network analysis to explored the influencing factors of shrinking cities from the perspective of economic, social and education. The topic is innovative, while there also have some problems should be revised as follow.

Author Response:

I appreciate your meaningful suggestions on our manuscripts. The details of the revision are as follows.

Comment No.1 (Reviewer #2)

1.There are lack of the theory or logic framework to illustrate the relationship of shrinking cities with the indicators. The author should not only calculated the correlation of them.

Author Response:

I appreciate your meaningful comments on our logical framework. The logical framework is urban modeling using various indicators of shrinking cities. That is because that the relationship does not occur through a simple linear mechanism but through a non-linear and complex mechanism. I added the explanation in Introduction and Discussion Section.

Manuscript Revision:

Ln 25-26	For example, population decline correlates with various types of social problems, such as economic decline and in-creasing vacant houses [10].
Ln 37-42	This means that the relationship would not reveal deductively using a fixed framework, but inductively using a large set of indicators. The relationships between these factors also do not occur through a simple linear mechanism, but through a non-linear and complex mechanism [12]. To understand these relationships, the logical framework to be utilized is an inductive statistical urban model using various indicators of shrinking cities [13]. The statistical urban models allow policymakers to monitor population decline [14].
Ln 193-194	The statistical urban model of this study indicates the cascading and complex relationships between the factors affecting population decline in shrinking cities.
Ln 274-275	10. Park Y, Newman GD, Lee J-E, Lee S. Identifying and comparing vacant housing determinants across South Korean cities. Appl Geography. 2021;136: 102566. doi:10.1016/j.apgeog.2021.102566
Ln 278-286	12. Baum-Snow N, Ferreira F. Causal inference in urban and regional economics. 2015;5: 3–68. doi:10.1016/B978-0-444-59517-1.00001-5<br>13. Kertész J, Török J, Murase Y, Jo H-H, Kaski K. Modeling the complex network of social interactions. In: Rudas T, Péli G, editors. Pathways between social science and computational social science. Cham: Springer; 2021. https://doi.org/10.1007/978-3-030-54936-7_1<br>14. Banzhaf E, Kindler A, Haase D. Monitoring and modeling indicators for urban

	shrinkage - the city of Leipzig, Germany. In: Center for Remote Sensing of Land Surfaces, editor. Second Workshop of the EARSeL SIG on Remote Sensing of Land Use & Land Cover - Application and Development. Bonn: Center for Remote Sensing of Land Surfaces; 2006. pp. 299–306.

Comment No.2 (Reviewer #2)

2. There should have some formulas or models in methods.

Author Response:

I appreciate your comments on the method section. According to your suggestion, I added some formulas about the PCR, STE, and MI.

Manuscript Revision:

Ln 29

$$PCR = \frac{P_{2010} - P_{2005}}{P_{2005}} \times 100 \quad (1)$$

Ln 119-120 T That is, TE is the derivative of the total effects curve computed at the a priori mean of that variable, $\delta_x = 0$ and $\delta_y = 0$ [33].

Ln 120-123 The standardized total effect (STE) normalized the TE by taking into account the ratio between the standard deviations of the indicators (σ_x) and the target indicator (PCR) (σ_y) [29]. The STE was calculated using Equation (2), as follows:

$$STE = \frac{\delta_y}{\delta_x} \times \frac{\sigma_y}{\sigma_x} \quad (2)$$

Ln 131-132 The MI was calculated in Equation (3), which is equivalent to Equation (4).

Ln 125-127 Correlation analysis was based on mutual information (MI), defined as the difference between the marginal entropy H(Y) of the target indicator (PCR) and its conditional entropy H(Y|X). The MI was calculated using Equation (3), which is equivalent to Equation (4); in the latter, $p(x, y)$ is the joint probability function of X and Y, while $p(x)$ and $p(y)$ are the marginal probability functions of X and Y.

Ln 137-138

$$MI\ (Y, X) = H\ (Y) - H\ (Y|X) \quad (3)$$

$$MI\ (T, X) = \sum_{x \in X} \sum_{y \in Y} p(x, y) \log_2 \frac{p(x, y)}{p(x)p(y)} \quad (4)$$

Comment No.3 (Reviewer #2)

3.The websites of data source should not only be added in references, but also needed to be added in data source.

Author Response:

I appreciate your comments on our data. As I commented in the data availability statement, the dataset

cannot be shared publicly because of governments' confidentiality. However, the dataset is available from the Japanese MEXT Institutional data access for researchers who meet the criteria for access to confidential data. Under the restriction, all indicators are listed in the supporting information file (S1 Table). The revision made it easy to understand the dataset.

Manuscript Revision:

Ln 91-96	This study used this dataset because it covers most publicly available city-level government statistics in Japan. Specifically, it stems from sources such as the Japanese Census, the School Basic Survey, the National Survey of Academic Progress, the Social Education Survey, the Survey on Time Uses and Leisure Activities, the Housing and Land Survey of Japan, the Major Financial Indicators of Local Governments, the Economic Census, and the National Survey of Family Income and Expenditure [29]. While this dataset cannot be shared publicly because of governments' confidentiality, the dataset is available from the Japanese MEXT Institutional data access for researchers who meet the criteria for access to confidential data.
Ln 97-98	All indicators are listed in S1 Table.

Comment No.4 (Reviewer #2)

4.In line 112, the authors mentioned population change rate. Is it refer to average population change rate in ten years or total population change rate in ten years.

Author Response:

I appreciate your meaningful comments on the definition of the population change rate. I mentioned the PCR in the Background sub-Section. However, I did not explain the meaning of PCR. I added the explanation in the Background Section.

Manuscript Revision:

Ln 21-22	In Fig 1, PCR is the percentage of change from population in 2005 (P2005) to population in 2010 (P2010), as calculated in Equation (1).
Ln 29	$PCR = \frac{P_{2010} - P_{2005}}{P_{2005}} \times 100 \quad (1)$

Comment No.5 (Reviewer #2)

5.In line 125, the authors illustrated there have 208 out of 259 indicators formed a significant network. However, there only have 14 indicators with STE more than 0.2. The author should listed all of correlation of 208 indicators.

Author Response:

I appreciate your meaningful comments on the STE score. As your suggestion, I added the supplement file in STE score of all indicators. The supplement file data indicates all of correlation of 208 indicators.

Manuscript Revision:

Ln 153-154 The STE, MI, and p-value of all indicators are listed in S1 Table.

Supplement file

Comment No.6 (Reviewer #2)

6.What's the difference of section bayesian network of shrinking cities with correlation of indicatiors. Maybe these two sections could be integrated into one section.

Author Response:

I appreciate your comments on the Result Section. According to your comments, I integrated the two sub-Section.

Manuscript Revision:

Ln 135 ~~Bayesian Network of Shrinking Cities~~ (Remove)

Ln 152 ~~Correlation of Indicators~~ (Remove)

Comment No.7 (Reviewer #2)

7.The significance of correlation should be mentioned. Such as 99%,95% and so on.

Author Response:

I appreciate your meaningful comments on significance of correlation. According to your suggestion, I added the p-value in Table 1 and supplement file. The Table 1 and supplement file indicates the significance of correlation of all indicators.

Manuscript Revision:

Ln 152 Table 1 shows the STE, MI, and BMI, and p-value of each indicator in relation to the PCR.

Ln 173 Table 1. STE, MI, and BMI, and p-value of each indicator.

Table 1

Indicators	STE	MI	BMI				p-value
			≤0	≤-4.0	≤-7.7	≤-12.6	
Population change rate aged 0-14	0.73	0.54	0.09	0.19	0.16	0.35	**
Population aged over 65	-0.70	0.50	0.08	0.16	0.14	0.35	**
Natural population change rate	0.57	0.30	0.05	0.10	0.05	0.22	**
Percentage of population aged 0-14	0.51	0.22	0.04	0.08	0.02	0.15	**
Number of children per teacher in elementary school	0.47	0.20	0.03	0.07	0.03	0.15	**
Average age of unmarried men	-0.43	0.16	0.03	0.06	0.01	0.11	**
Number of children per teacher in junior high school	0.36	0.10	0.02	0.04	0.01	0.08	**
Population change rate aged over 65	0.30	0.08	0.01	0.03	0.01	0.06	**
Financial strength index	0.29	0.09	0.01	0.04	0.01	0.07	**
Percentage of combined classes in elementary school	-0.27	0.06	0.01	0.03	0.00	0.05	**
Average age of unmarried women	-0.26	0.05	0.01	0.02	0.00	0.04	**
Number of students per educational computer in junior high schools	0.25	0.06	0.01	0.02	0.00	0.05	**
Number of students per educational computer in schools	0.21	0.04	0.01	0.02	0.00	0.03	**
Percentage of workers in primary industry	-0.20	0.04	0.01	0.01	0.00	0.03	**

Note: STE, Standardized Total Effect; MI, Mutual Information; BMI, Binary Mutual Information; *, $p < 0.05$; **, $p < 0.01$

Supplement file

Comment No.8 (Reviewer #2)

8.In line 51, the authors could re-draw a new figure of study area to show the 1316 cities clearly by

GIS. The color of study area and non-study area are different.

Author Response:

I appreciate your comments on Fig 1. As you commented, the distinction between the study and non-study areas is confusing. Therefore, I highlighted the outer frame of the study areas. In addition, I added a figure focusing on a specific metropolitan area. The revision made it easy to understand the study areas.

Manuscript Revision:

Figure 1

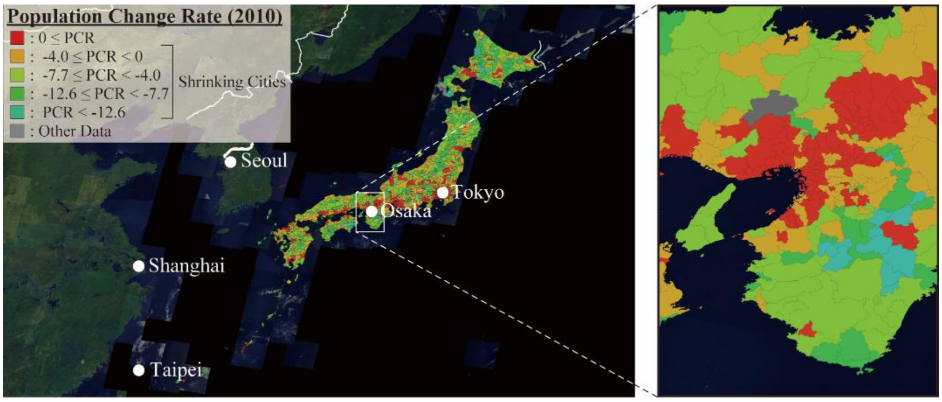

Comment No.9 (Reviewer #2)

9.In table 1, what's the BMI refer to in different ranges? It should be explained clearly.

Author Response:

I appreciate your comments on the meaning of BMI. As I mentioned in the previous manuscript, BMI is the amount of information brought by each indicator to the knowledge of the state of the target indicator (PCR) compared to an unconnected network. According to your comment, I added the explanation of the meaning of BMI with equations; BMI is the amount of information brought by each variable to the knowledge of the state of the target indicator (PCR). In addition, I added the note at the bottom of Table 1.

Manuscript Revision:

Ln 131-132 That means that BMI is amount of information brought by each variable to the knowledge of the state of the target indicator (PCR).

Ln 128-129

$$MI\ (T, X) = H\ (T) - H\ (T|X) \quad (3)$$

$$MI\ (T, X) = \sum_{x \in X} \sum_{y \in Y} p(x, y) \log_2 \frac{p(x, y)}{p(x)p(y)} \quad (4)$$

Table 1 STE, Standardized Total Effect; MI, Mutual Information; BMI, Binary Mutual Information.

Comment No.10 (Reviewer #2)

10.In conclusion, the author should illustrate some conclusions in your own research. What can be concluded by your research? It is not suitable to cite others researches.

Author Response:

I appreciate your comments on the conclusion. According to your suggestion, I removed references and concluded my main conclusions sharply.

Manuscript Revision:

Ln 243-253	In conclusion, the study findings provide essential insights into the importance of education investment for children in shrinking cities. Regarding theoretical implications, this study shows that the impact of educational in-vestment is more substantial than that of economic indicators (e.g., the financial strength index). With this in mind and considering the limitations regarding fiscal expenditures in Japan, it may be important for municipal leaders to invest in education for children in order to prevent rapid population decline. In summary, increasing the budget for education to tackle the decline in the number of children and increasing the welfare expenditure to tackle the growth in the number of older adults are likely important measures in the context of population decline because of low birth rates and aging populations.<br>In Japan, population decline is expected to continue for a long time, as immigration is low. In this context, Japanese shrinking cities need to achieve a gradual, rather than a rapid, population decline to maintain residents' abundant lifestyle. The conclusions of this study could contribute to countries other than Japan that are likely to experience future population decline because of low birth rates and aging populations.

Comment No.11 (Reviewer #2)

11.The serial number of sub-title should be added.

Author Response:

I appreciate your comments on the manuscript format. According to your suggestion, I added the serial number of the sub-title.

Manuscript Revision:

Ln 13	1.1 Background
Ln 45	1.2 Purpose
Ln 60	1.3 Literature Review
Ln 88	2.1 Economic, Social, and Educational Indicators
Ln 104	2.2 Bayesian Network Analysis

General Comment (Editor)

When submitting your revision, we need you to address these additional requirements.

Author Response:

I appreciate your technical suggestions regarding manuscript style, data availability, and CC BY 4.0 license. I respond to those three points. The revisions were as follows:

Comment No.1(Editor)

1. Please ensure that your manuscript meets PLOS ONE's style requirements, including those for file naming. The PLOS ONE style templates can be found at https://journals.plos.org/plosone/s/file?id=wjVg/PLOSOne_formatting_sample_main_body.pdf and https://journals.plos.org/plosone/s/file?id=ba62/PLOSOne_formatting_sample_title_authors_affiliations.pdf

Author Response:

I appreciate your comments regarding the format of the manuscript. I revised the manuscript to meet PLOS ONE's style requirements. The revisions were all of the manuscripts. Therefore, I want you to check the revised manuscript.

Manuscript Revision:

Ln 352-354 Supporting information

S1 Table. Results of the Economic, Social, and Educational Indicators. The table below shows the 259 indicators with their average, standard deviation score, standardized total effect, mutual information, and p-value.

Ln 173-176 Table 1. STE, MI, BMI, and p-value of each indicator. Table 1 focuses on 14 indicators with STE more than |0.2|. In Table 1, STE is standardized total effects. MI is mutual information. BMI is binary mutual information. * indicates p-value < 0.05. ** indicates p-value < 0.01.

			BMI				
Indicators	STE	MI	≤0	≤-4.0	≤-7.7	≤-12.6	p-value
Population change rate aged 0-14	0.73	0.54	0.09	0.19	0.16	0.35	**
Population aged over 65	-0.70	0.50	0.08	0.16	0.14	0.35	**
Natural population change rate	0.57	0.30	0.05	0.10	0.05	0.22	**
Percentage of population aged 0-14	0.51	0.22	0.04	0.08	0.02	0.15	**
Number of children per teacher in elementary school	0.47	0.20	0.03	0.07	0.03	0.15	**
Average age of unmarried men	-0.43	0.16	0.03	0.06	0.01	0.11	**
Number of children per teacher in junior high school	0.36	0.10	0.02	0.04	0.01	0.08	**
Population change rate aged over 65	0.30	0.08	0.01	0.03	0.01	0.06	**
Financial strength index	0.29	0.09	0.01	0.04	0.01	0.07	**
Percentage of combined classes in elementary school	-0.27	0.06	0.01	0.03	0.00	0.05	**
Average age of unmarried women	-0.26	0.05	0.01	0.02	0.00	0.04	**
Number of students per educational computer in junior high schools	0.25	0.06	0.01	0.02	0.00	0.05	**
Number of students per educational computer in schools	0.21	0.04	0.01	0.02	0.00	0.03	**
Percentage of workers in primary industry	-0.20	0.04	0.01	0.01	0.00	0.03	**

Comment No.2(Editor)

2. In your Data Availability statement, you have not specified where the minimal data set underlying the results described in your manuscript can be found. PLOS defines a study's minimal data set as the underlying data used to reach the conclusions drawn in the manuscript and any additional data required to replicate the reported study findings in their entirety. All PLOS journals require that the minimal data set be made fully available. For more information about our data policy, please see http://journals.plos.org/plosone/s/data-availability.<br>Upon re-submitting your revised manuscript, please upload your study's minimal underlying data set as either Supporting Information files or to a stable, public repository and include the relevant URLs, DOIs, or accession numbers within your revised cover letter. For a list of acceptable repositories, please see http://journals.plos.org/plosone/s/data-availability#loc-recommended-repositories. Any potentially identifying patient information must be fully anonymized.<br>Important: If there are ethical or legal restrictions to sharing your data publicly, please explain these restrictions in detail. Please see our guidelines for more information on what we consider unacceptable restrictions to publicly sharing data: http://journals.plos.org/plosone/s/data-availability#loc-unacceptable-data-access-restrictions. Note that it is not acceptable for the authors to be the sole named individuals responsible for ensuring data access.<br>We will update your Data Availability statement to reflect the information you provide in your cover letter.

Author Response:

I appreciate your comments on the data availability. I wrote the data availability statement in the system. In addition, I uploaded the minimal underlying data set as supporting information files. According to your comments, I added the explanation in the manuscript. I would you like to check the statement.

Manuscript Revision:

Ln 95-96	While this dataset cannot be shared publicly because of governments' confidentiality, the dataset is available from the Japanese MEXT Institutional data access for researchers who meet the criteria for access to confidential data.
Ln 97-98	All indicators are listed in S1 Table.
Ln 153-154	The STE, MI, and p-value of all indicators are listed in S1 Table.

Comment No.3(Editor)

3. We note that Figure 1 in your submission contain [map/satellite] images which may be copyrighted. All PLOS content is published under the Creative Commons Attribution License (CC BY 4.0), which means that the manuscript, images, and Supporting Information files will be freely available online, and

any third party is permitted to access, download, copy, distribute, and use these materials in any way, even commercially, with proper attribution. For these reasons, we cannot publish previously copyrighted maps or satellite images created using proprietary data, such as Google software (Google Maps, Street View, and Earth). For more information, see our copyright guidelines: http://journals.plos.org/plosone/s/licenses-and-copyright.

We require you to either (1) present written permission from the copyright holder to publish these figures specifically under the CC BY 4.0 license, or (2) remove the figures from your submission:

a. You may seek permission from the original copyright holder of Figure 1 to publish the content specifically under the CC BY 4.0 license.

We recommend that you contact the original copyright holder with the Content Permission Form (http://journals.plos.org/plosone/s/file?id=7c09/content-permission-form.pdf) and the following text:

“I request permission for the open-access journal PLOS ONE to publish XXX under the Creative Commons Attribution License (CCAL) CC BY 4.0 (http://creativecommons.org/licenses/by/4.0/). Please be aware that this license allows unrestricted use and distribution, even commercially, by third parties. Please reply and provide explicit written permission to publish XXX under a CC BY license and complete the attached form.”

Please upload the completed Content Permission Form or other proof of granted permissions as an "Other" file with your submission.

In the figure caption of the copyrighted figure, please include the following text: “Reprinted from [ref] under a CC BY license, with permission from [name of publisher], original copyright [original copyright year].”

b. If you are unable to obtain permission from the original copyright holder to publish these figures under the CC BY 4.0 license or if the copyright holder’s requirements are incompatible with the CC BY 4.0 license, please either i) remove the figure or ii) supply a replacement figure that complies with the CC BY 4.0 license. Please check copyright information on all replacement figures and update the figure caption with source information. If applicable, please specify in the figure caption text when a figure is similar but not identical to the original image and is therefore for illustrative purposes only.

The following resources for replacing copyrighted map figures may be helpful:

USGS National Map Viewer (public domain): http://viewer.nationalmap.gov/viewer/

The Gateway to Astronaut Photography of Earth (public domain): http://eol.jsc.nasa.gov/sseop/clickmap/

Maps at the CIA (public domain): https://www.cia.gov/library/publications/the-world-factbook/index.html and https://www.cia.gov/library/publications/cia-maps-publications/index.html

NASA Earth Observatory (public domain): http://earthobservatory.nasa.gov/

Landsat: http://landsat.visibleearth.nasa.gov/

USGS EROS (Earth Resources Observatory and Science (EROS) Center) (public domain):

http://eros.usgs.gov/#
Natural Earth (public domain): http://www.naturalearthdata.com/

Author Response:

I appreciate your helpful suggestions regarding the figure copyright. I suppled a replacement figure of Landsat (http://landsat.visibleearth.nasa.gov/) that complies with the CC BY 4.0 license. In addition, I update the figure caption with source information.

Manuscript Revision:

Fig.1-

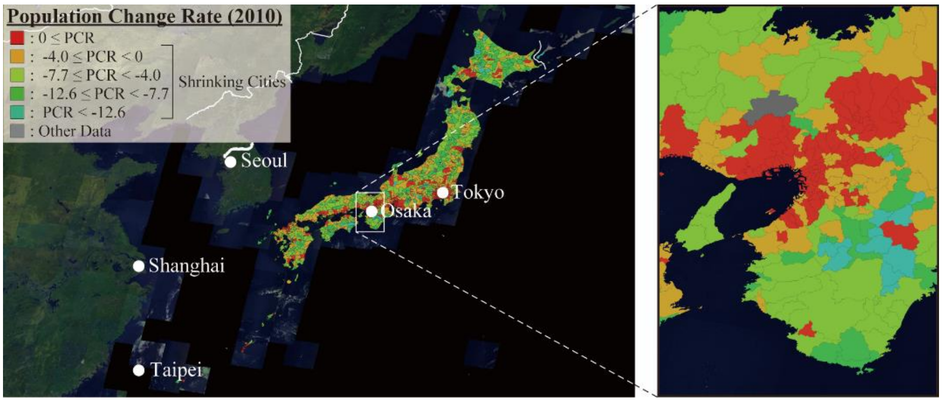

Ln 31-34 Fig 1. Population change rate in Japanese cities in 2010. In Fig 1, left side map indicates the location of Tokyo and Osaka in major East Asian cities, and right side indicates map focused on the Osaka Metropolitan area. Fig 1 shows the population change in graduated colors from red to blue. The gray color areas are other cities, such as merged cities. Republished from Fig.1 under a CC BY license, with permission from landsat, original copyright 2023.

Again, thank you for giving us the opportunity to strengthen our manuscript with your valuable comments. I have worked hard to incorporate your feedback and hope that these revisions persuade you to accept our submission.

Sincerely,
