# Supplementary material for: Urban modeling of shrinking cities through Bayesian network analysis using economic, social, and educational indicators: Case of Japanese cities
Source: PLoS One. 2023 Apr 10;18(4):e0284134. doi: 10.1371/journal.pone.0284134 (PMC10085021; doi:10.1371/journal.pone.0284134)
Supplement: Attachment — Submitted filename: 3_Response to Reviewers_20230319.pdf [file pone.0284134.s003.pdf]

PONE-D-22-35408

Dear Reviewer

Haruka KATO

I appreciate the time and effort you have dedicated to providing insightful feedback on strengthening my manuscript. Thus, it is with great pleasure that I re-submit my article for further consideration. I have incorporated changes that reflect the detailed suggestions you have graciously provided. I also hope that my edits and the responses I provide below satisfy the issue and concern you have noted. The revised manuscript is marked with tracked change.

General Comment (Reviewer #2)

The quality of this manuscript have improved after revision. There only have two problems should be revised.

Author Response:

I appreciate your evaluation and meaningful comments on our manuscripts. According to your comments, I revised the manuscript as follows:

Comment No.1 (Reviewer #2)

1.The authors choose population change rate aged 0-14 and population aged over 65. Why the authors not choose age 15-64. It should be explained simply.

Author Response:

I appreciate your comments on the results of the Bayesian network analysis. As you suggested, Table 1 does not choose the 'population aged 15-65' but the 'population aged over 65' and the 'population change rate aged 0-14'. That is because the population decline in Japanese shrinking cities occurred primarily due to natural population change rather than population change because of migration. In detail, the Bayesian network shows the weak relationship between the "population aged 15-65" and the PCR. I have simply added the explanation in the manuscript.

Manuscript Revision:

Ln 160-165	It is worth noting the low STE of the "population aged 15-65" (STE=0.09), which was not included in Table 1. The strong relationship between the "population aged 15-65" and the PCR might suggest that the population has decreased due to migration, as in other shrinking cities in the US and European countries. However, the model indicates the weak relationship between the "population aged 15-65" and the PCR in Japanese shrinking cities.

Comment No.2 (Reviewer #1)

2.Some relevant references should be cited.

Spatial evolution of population change in Northeast China during 1992–2018. Science of the Total Environment.2021,776:146023.https://doi.org/10.1016/j.scitotenv.2021.146023.<br>Spatial and temporal heterogeneity of urban land area and PM2.5 concentration in China.Urban Climate,2022,45:101268.doi:https://doi.org/10.1016/j.uclim.2022.101268.<br>Spatial-Temporal Characteristics of Primary and Secondary Educational Resources for Relocated Children of Migrant Workers:The Case of Liaoning Province.Complexity,volume 2020,Article ID7457109,13 pages.doi:https://doi.org/10.1155/2020/7457109.<br>Theoretical framework and research prospect of the impact of China's digital economic development on population.Frontiers in Earth Science,2022,10:988608. doi: 10.3389/feart.2022.988608.<br>Differences in Accessibility of Public Health Facilities in Hierarchical Municipalities and the Spatial Pattern Characteristics of Their Services in Doumen District,China.Land 2021, 10, 1249. https://doi.org/10.3390/land10111249.<br>Spatiotemporal relationship characteristic of climate comfort of urban human settlement environment and population density in China. Front. Ecol. Evol. 2022,10:953725.doi: 10.3389/fevo.2022.953725.<br>Spatio–temporal evolution and factors of climate comfort for urban human settlements in the Guangdong–Hong Kong–Macau Greater Bay Area. Front. Environ. Sci. 2022,10:1001064. doi: 10.3389/fenvs.2022.1001064

Author Response:

I appreciate your comment on the additional reference papers. As you suggested, I added some relevant references regarding the statistical analysis of population change, which reviewer No.2 suggested. This revision reinforces the logic of the manuscript.

Manuscript Revision:

Ln 273-274	7. You H, Yang J, Xue B, Xiao X, Xia J, Jin C, et al. Spatial evolution of population change in Northeast China during 272 1992–2018. Science of The Total Environment. 2021;776: 146023. doi:10.1016/j.scitotenv.2021.146023
Ln 350-352	42. Zhou C, Zhang D, Cao Y, Wang Y, Zhang G. Spatio–temporal evolution and factors of climate comfort for urban human settlements in the Guangdong–Hong Kong–Macau Greater Bay Area. Front Environ Sci. 2022;10. doi:10.3389/fenvs.2022.1001064

Again, thank you for giving us the opportunity to strengthen our manuscript with your valuable comments. I have worked hard to incorporate your feedback and hope that these revisions persuade you to accept our submission.

Sincerely,
